# Coinfection with EBV/CMV and other respiratory agents in children with suspected infectious mononucleosis

**DOI:** 10.1186/1743-422X-7-247

**Published:** 2010-09-21

**Authors:** Xia Wang, Kun Yang, Cong Wei, Yuan Huang, Dongchi Zhao

**Affiliations:** 1 Pediatrics Department, Zhongnan Hospital, Wuhan University, Wuhan 430071, China; 2 The Sixth People's Hospital of Hangzhou, Hangzhou Children's Hospital, Hangzhou, China

## Abstract

**Background:**

Numerous studies have shown that Epstein-Barr virus (EBV) and cytomegalovirus (CMV) can infect immunocompetent patients simultaneously with other agents. Nonetheless, multiple infections with other agents in EBV/CMV-infected children have received little attention. We conducted a retrospective study of children with suspected infectious mononucleosis. Peripheral blood samples were analyzed by indirect immunofluorescence to detect EBV, CMV and other respiratory agents including respiratory syncytial virus; adenovirus; influenza virus types A and B; parainfluenza virus types 1, 2 and 3; *Chlamydia pneumonia*e and *Mycoplasma pneumoniae*. A medical history was collected for each child.

**Results:**

The occurrence of multipathogen infections was 68.9%, 81.3% and 63.6% in the children with primary EBV, CMV or EBV/CMV, respectively, which was significantly higher than that in the past-infected group or the uninfected group (*p *< 0.001). Of the multipathogen-infected patients, the incidence of *C. pneumoniae *in children with primary infection was as high as 50%, significantly higher than in the other groups (*p *< 0.001). In the patients with multipathogen infection and EBV/CMV primary infection, fever, rash, lymphadenopathy, hepatomegaly, splenomegaly, atypical lymphocytes and abnormal liver function were more frequent and the length of hospital stay and duration of fever were longer than in other patients.

**Conclusion:**

Our study suggests that there is a high incidence of multipathogen infections in children admitted with EBV/CMV primary infection and that the distribution of these pathogens is not random.

## Introduction

Epstein-Barr virus (EBV) and Cytomegalovirus (CMV), members of the herpesvirus family, are common viruses that cause infectious mononucleosis (IM) characterized by fever, pharyngitis and lymphadenopathy. EBV/CMV infects at least 90% of the world's population and can persist in a latent form after primary infection. Reactivation can occur years later, particularly under conditions of immunosuppression [1,2]. The primary infection may occur shortly after the disappearance of maternal antibodies during infancy [3]. In childhood, EBV is the most common cause of IM, but primary CMV infection will cause up to 7% of cases of mononucleosis syndrome and will manifest symptoms almost indistinguishable from those of EBV-induced mononucleosis [4].

It is well known that EBV and CMV are common opportunistic infection agents in the immunocompromised, including human immunodeficiency virus-infected individuals, and are a major source of serious viral complications in organ transplant recipients [5]. Children are also a susceptible population at high risk of CMV/EBV infection. During growth and development, CMV/EBV infection can depress the host immune response: this is a major cause of recurrent childhood microbial infection [6]. Because CMV and EBV have so much in common, coinfection with these two viruses occurs occasionally in children [7-9]. Numerous studies have shown that EBV/CMV can infect immunocompetent patients simultaneously with other agents including respiratory syncytial virus (RSV), *Chlamydia pneumoniae *(CP), human herpesvirus 6, measles virus and others[7,10-14], and it has been reported that EBV/CMV-infected children with no detected immune deficiency can suffer from mixed infections with other agents[12,14]. In a previous study, we found that multipathogen infection is not random but is related to specific agents. Nonetheless, multiple infections of EBV/CMV and other agents have received little attention. The aim of this study was to explore the clinical features and incidence of coinfection of EBV/CMV and respiratory pathogens in children hospitalized with suspected IM.

## Results

### Clinical features

#### EBV infection

Of the 190 patients, 164 had detectable EBV antibodies. The age range of this group was from 1-164 months (mean 46.9 ± 35.7 months) with a male: female ratio of 1.73:1 (102 boys and 62 girls). Forty patients had primary EBV infection, 48 past infection and 76 were uninfected. The clinical characteristics of these three groups are presented in Table 1. There were no differences between the groups in incidence of fever, rash, palatal petechiae or splenomegaly, but the mean hospital stay in the past-infected group was the shortest (7.71 ± 3.07 days). The patients with EBV primary infection had a higher incidence of lymphadenopathy than the other two groups (*p* < 0.001). In the primary-infection and past-infected groups pharyngitis and hepatomegaly were more frequent than in uninfected patients (*p *= 0.02 and 0.013, respectively). There were no differences between these three groups in their main laboratory results, except that the percentage of patients with > 10% atypical lymphocytes was higher in the primary- and past-infected groups than in the uninfected group and the frequency of C-reactive protein (CRP) > 10 mg/L was significantly lower in the primary-infection group.

**Table 1:** The main clinical features in patients grouped by EBV detection.

Clinical features	primary infected (n = 40)	past infected (n = 48)	uninfected (n = 76)
Age	8-164 months	2-163 months	1-140 months
1-12 months	3 (7.50%)	7 (14.6%)	20 (26.3%)
12-36 months	17 (42.5%)	11 (22.9%)	23 (30.3%)
36-72 months	8 (20.0%)	17 (35.4%)	21 (27.6%)
> 72 months	12(30.0%)	13(27.1%)	12(15.8%)
Sex, male/female	20/20	20/18	52/24
Length of stay, days	9.53 ± 3.52*	7.71 ± 3.07**	9.11 ± 4.11*
Duration of fever, days	6.43 ± 4.21	6.04 ± 4.19	4.99 ± 4.67
Fever	36 (90%)	42 (87.5%)	64 (84.2%)
Rash	8 (20.0%)	9 (18.8%)	13 (17.1%)
Lymphadenopathy	24 (60.0%)*	14 (29.2%)**	29 (38.2%)**
Pharyngitis	39 (97.5%)	45 (93.8%)	75 (98.7%)
Palatal petechiae	9 (22.5%)	13 (27.1%)	16 (21.1%)
Hepatomegaly	8 (20.0%)*	9 (18.8%)*	7 (9.21%)**
Splenomegaly	4 (10.0%)	3 (6.25%)	4 (5.26%)
ALC < 10%	10/27 (37.0%)*	11/26 (42.3%)**	11/46 (23.9%)*
Elevated ESR	16/28 (57.1%)	18/31 (58.1%)	19/43 (44.2%)
CRP > 10 mg/L	13/26 (50.0%)*	22/33 (66.7%)**	31/48 (64.6%)**
ALF	7/22 (31.8%)	5/18 (27.8%)	10/24 (41.7%)
WBC count, 10^9^/L	11.94 ± 8.58	10.20 ± 5.67	10.47 ± 5.99
Neutrophils, %	40.48 ± 24.43	49.07 ± 21.81	41.99 ± 26.24
Lymphocytes, %	48.37 ± 23.65	39.86 ± 22.03	45.65 ± 25.58
Monocytes, %	9.98 ± 6.12	9.58 ± 4.61	9.86 ± 6.26
Platelets, 10^9^/L	263.61 ± 125.37	286.38 ± 142.72	288.90 ± 130.82
Hemoglobin, g/L	116.53 ± 8.85	117.68 ± 10.83	117.90 ± 10.23

Between * and **, the *p *value < 0.05. ALC: atypical lymphocytes; ESR: erythrocyte sedimentation rate; CRP: C-reactive protein; ALF: abnormal liver function (alanine aminotransferase or aspartate aminotransferase higher than 46 U/L); WBC: white blood cell.

#### CMV infection

Of the 190 patients, 165 had the test for CMV-specific antibodies, including 106 boys and 59 girls (a male:female ratio of 1.80:1) with ages ranging from 1-164 months (mean 43.5 ± 35.4 months). Twenty-five patients had primary CMV infection, 104 were past-infected and 36 uninfected. Compared with the other two groups, the primary-infection group had a longer hospital stay and more frequent presentation of palatal petechiae, hepatomegaly and splenomegaly, atypical lymphocytes > 10% and abnormal liver function, but fewer rashes. Although the total numbers of white blood cells (WBC), platelet and hemoglobin values did not differ among groups, the primary-infected children had the lowest percentage of neutrophils (24.15 ± 15.70%, *p *= 0.001) and the highest percentage of lymphocytes (62.03 ± 16.74%, *p *= 0.003). No parameter differed significantly between the past-infected and uninfected groups (Table 2).

**Table 2:** The main clinical features in CMV-detected groups.

Clinical features	primary infected (n = 25)	past infected (n = 104)	uninfected (n = 36)
Age	1-110 months	2-164 months	4-163 months
1-12 months	7 (28.0%)	22 (21.2%)	12 (30.6%)
12-36 months	8 (32.0%)	36 (34.6%)	11 (36.1%)
36-72 months	6 (24.0%)	25 (24.0%)	6 (16.7%)
> 72 months	4 (16.0%)	21 (20.2%)	6 (16.7%)
Sex, male/female	17/8	65/39	24/12
Length of stay, days	13.04 ± 4.16*	8.26 ± 3.07**	8.28 ± 4.14**
Duration of fever, days	5.36 ± 4.32	4.96 ± 4.38	6.03 ± 5.11
Fever	20 (80.0%)	82 (78.8%)	29 (80.6%)
Rash	1 (4.00%)*	24 (23.1%)**	7 (19.4%)**
Lymphadenopathy	13 (52.0%)	37 (35.6%)	9 (25%)
Pharyngitis	23 (92.0%)	101 (97.1%)	34 (94.4%)
Palatal petechiae	10 (40.0%)*	16 (15.4%)**	2 (5.56%)**
Hepatomegaly	13 (52.0%)*	12 (11.5%)**	2 (5.56%)**
Splenomegaly	8 (32.0%)*	3 (2.88%)**	1 (2.78%)**
ALC > 10%	12/16 (75.0%)*	12/58 (20.7%)**	4/14 (28.6%)**
Elevated ESR	8/14 (57.1%)	35/61 (57.4%)	13/21 (61.9%)
CRP > 10 mg/L	8/15 (53.3%)	33/61 (54.1%)	16/22 (72.7%)
ALF	15/21 (71.4%)*	9/37 (24.3%)**	3/13 (23.1%)**
WBC count, 10^9^/L	14.42 ± 8.31	11.07 ± 15.72	9.07 ± 6.14
Neutrophils, %	24.15 ± 15.70*	43.60 ± 23.3**	39.44 ± 25.90**
Lymphocytes, %	62.03 ± 16.74*	44.79 ± 22.39**	48.92 ± 25.57**
Monocytes, %	10.63 ± 5.81	9.89 ± 6.36	9.63 ± 4.90
Platelets, 10^9^/L	253.96 ± 96.02	304.72 ± 143.25	305.97 ± 121.85
Hemoglobin, g/L	113.30 ± 9.91	118.59 ± 11.92	118.35 ± 12.26

Between * and **, the *p *value < 0.05. ALC: atypical lymphocytes; ESR: erythrocyte sedimentation rate; CRP: C-reactive protein; ALF: abnormal liver function (alanine aminotransferase or aspartate aminotransferase higher than 46 U/L); WBC: white blood cell.

#### EBV or CMV infection and clinical features

**Figure 1:**
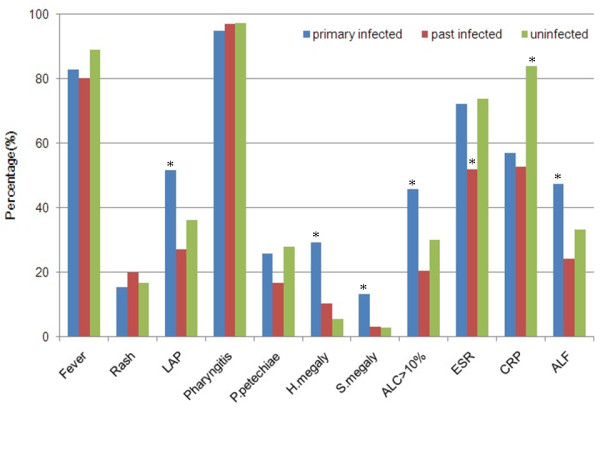
**Main clinical features in patients grouped by detection of anti-EBV or anti-CMV antibodies**. *Differs from the other two groups, *p *< 0.05. LAP: lymphadenopathy; P. petechiae: palatal petechiae; H.megaly: hepatomegaly; S.megaly: splenomegaly; ALC: atypical lymphocytes; ESR: erythrocyte sedimentation rate; CRP: C-reactive protein; ALF: abnormal liver function (alanine aminotransferase or aspartate aminotransferase higher than 46 U/L).

Patients were classified into three groups. Group A included 58 patients who had primary infection with EBV or CMV, group B consisted of 96 patients with past infection with EBV or CMV and group C consisted of 36 patients uninfected with EBV or CMV. The clinical features of these groups are shown in Fig. 1. Compared with groups B and C, group A had longer hospital stays and lymphadenopathy, hepatomegaly, splenomegaly, atypical lymphocytes > 10% and abnormal liver function were more frequent. The proportion of patients with CRP > 10 mg/L was greater in group C than in the other two groups (*p *= 0.03). There were no differences between groups A, B and C in duration of fever, incidence of fever, rash, pharyngitis and palatal petechiae or elevated erythrocyte sedimentation rate (ESR).

In addition, seven children showed both EBV and CMV primary infection (Table 3). Of these, six were less than six years old. All seven patients showed the typical manifestations of IM--fever, pharyngitis and lymphadenopathy. Palatal petechiae, hepatomegaly and splenomegaly were each seen in four children (57.1%) and none presented with rashes. The occurrence of liver function abnormalities was 80% (4/5) and an elevation in the proportion of atypical lymphocytes was observed in five children (5/6, 83.3%). White blood cell counts ranged from 7.88 × 10^9^/L to 43.8 × 10^9^/L. Of the seven children, four had detectable specific IgM against one or more of the other 12 respiratory agents. The results showed one child positive for one type of IgM and the other three each positive for two types.

**Table 3:** Clinical features of the seven children with EBV and CMV primary infection.

Clinical features	Patients						
	
	N°1	N°2	N°3	N°4	N°5	N°6	N°7
Age, months	73	59	16	24	65	13	24
Sex	female	male	female	male	male	male	male
Fever	+	+	+	+	+	+	+
Lymphadenopathy	+	+	+	+	+	+	+
Pharyngitis	+	+	+	+	+	+	+
Palatal petechiae	-	+	+	+	+	-	-
Rash	-	-	-	-	-	-	-
Hepatomegaly	-	+	+	-	+	-	+
Splenomegaly	-	+	+	+	+	-	-
ALT, U/L	/	18	77	70	256	77	/
AST, U/L	/	28	84	56	54	78	/
WBC count, 10^9^/L	7.88	16.2	27.6	22.0	9.14	43.8	10.5
Lymphocyte, %	29.3	70.7	77.4	47.7	75.3	87.0	43.3
ALC, %	4	/	58	57	37	15	56
Other positive agents	CP, MP	/	MP	/	/	Adv, KP	Adv, MP

ALC: atypical lymphocytes; ALT: alanine aminotransferase; AST: aspartate aminotransferase; WBC: white blood cell; CP: *Chlamydia pneumoniae*; MP: *Mycoplasma pneumoniae*; KP: *Klebsiella pneumoniae*; Adv: adenovirus.

### The disease spectrum in children with EBV/CMV infection

The disease spectrum was diverse, especially the spectrum of EBV infection (Table 4). The most common disease caused by EBV primary infection was IM (21/40, 52.5%), followed by respiratory tract infection (12/40, 30.0%), Kawasaki disease (1/40, 2.5%), anaphylactic purpura (1/40, 2.5%), idiopathic thrombocytopenic purpura (1/40, 2.5%), measles (1/40, 2.5%), asthma (1/40, 2.5%), juvenile rheumatoid arthritis (1/40, 2.5%) and ulcerative stomatitis (1/40, 2.5%). Of the diseases caused by CMV primary infection, the most common was also IM (14/25, 56.0%), followed by respiratory tract infections (9/25, 36.0%).

**Table 4:** The disease spectrum in EBV or CMV primary infected children.

Diagnosis	EBV primary infected (n = 40)		CMV primary infected (n = 25)	
		
	N	Percentage (%)	N	Percentage (%)
IM	21	52.5	14	56.0
Respiratory infection	12	30.0	9	36.0
Kawasaki disease	1	2.5		
Anaphylactic purpura	1	2.5		
Measles	1	2.5		
Ulcerative stomatitis	1	2.5		
Asthma	1	2.5		
JRA	1	2.5		
ITP	1	2.5		
Hyperbilirubinemia			1	4.0
Infantile hepatitis			1	4.0

IM: infectious mononucleosis; ITP: idiopathic thrombocytopenic purpura; JRA: juvenile rheumatoid arthritis.

### Coinfection of EBV/CMV with other pathogens

**Figure 2:**
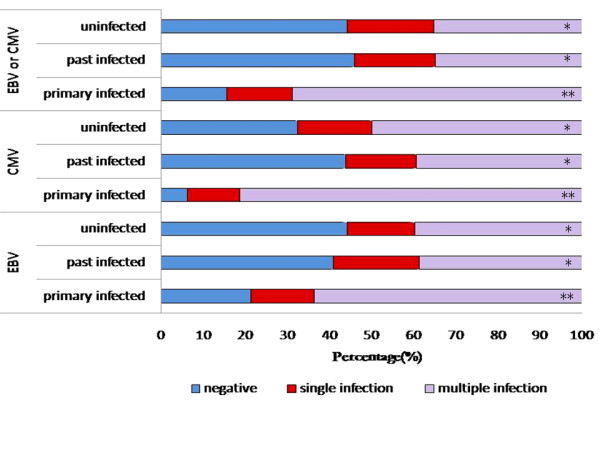
**Coinfection of EBV or CMV and other pathogens**. Between * and ** the *p *value < 0.01.

Besides EBV and CMV, 162 patients had detectable specific IgM against the other 12 pathogens RSV, Adv, Flu A and B, PIV 1, 2, and 3, CP, MP, *Haemophilus influenzae*, *Klebsiella pneumoniae *and *Legionella pneumophila*. Of these patients, 60 (37.0%) children were uninfected, a single agent was identified in 30 (18.5%) children and two or more agents in 72 (44.4%) children. Fig. 2 shows the details of coinfection with EBV or CMV and other pathogens. The general distribution of these 12 pathogens was similar in the patients with detectable anti-EBV, anti-CMV and anti-EBV or anti-CMV. We detected coinfection of multiple other agents and EBV/CMV in 68.9% of children, and in 63.6% of children with only anti-EBV or anti-EBV or anti-CMV. In the group with only anti-CMV antibodies detected, the proportion was higher at 81.3%, which differed significantly from the past-infected and uninfected groups.

The patients were divided into six groups based on the results of testing for antibodies to EBV or CMV and the other 12 pathogens (Table 5). We compared the clinical manifestations of these six groups. The symptoms and physical signs seemed to be most severe in the patients of group A (i.e., the patients with EBV or CMV primary infection and two or more other pathogens). In this group, fever, rash, lymphadenopathy, hepatomegaly, splenomegaly, atypical lymphocytes > 10% and abnormal liver function were all very frequent. In addition, the length of hospital stay and the duration of fever were longer than in groups C, D and F.

**Table 5:** The differences in the main clinical features of children with multiple infections or a single infection.

Clinical features	A(n = 31)	B(n = 14)	C(n = 29)	D(n = 54)	E(n = 12)	F(n = 22)
H. stay, days	10.87 ± 4.11**	10.07 ± 4.23	7.69 ± 3.24*	8.09 ± 3.15*	9.08 ± 3.23	7.73 ± 3.88*
D. of fever, days	7.39 ± 3.93**	5.79 ± 4.89	4.62 ± 4.07*	4.80 ± 4.44*	7.33 ± 5.63	4.32 ± 3.20*
Fever	30 (96.8%)**	11 (78.6%)*	22 (75.9%)*	43 (79.6%)*	11 (78.6%)*	19 (86.4%)*
Rash	7 (22.6%)	0 (0)	7 (24.1%)	9 (16.7%)	1 (8.33%)	5 (22.7%)
Lymphadenopathy	16 (51.6%)**	7 (50.0%)**	9 (31.0%)*	13 (24.1%)*	3 (25.0%)*	9 (40.9%)
Pharyngitis	31 (100%)	13 (92.9%)	28 (96.6%)	52 (96.3%)	11 (91.7%)	22 (100%)
Palatal petechiae	6 (19.4%)	4 (28.6%)	6 (20.7%)	8 (14.8%)	4 (33.3%)	4 (18.2%)
Hepatomegaly	7 (22.6%)*	3 (21.4%)*	3 (10.3%)**	4 (7.41%)**	0 (0)	2 (9.09%)**
Splenomegaly	3 (9.38%)	1 (7.41%)	1 (3.45%)	2 (3.70%)	0 (0)	1 (4.55%)
ALC > 10%	8/10 (80.0%)**	5/10 (50.0%)**	2/10 (20.0%)*	8/32 (25.0%)*	2/7 (28.6%)*	4/12 (33.3%)*
Elevated ESR	14/17 (82.4%)**	7/9 (77.8%)*	11/18 (61.1%)*	15/29 (51.7%)*	5/5 (100%)**	8/13 (61.5%)*
CRP > 10 mg/L	9/17 (52.9%)*	5/10 (50.0%)*	9/18 (50.0%)*	16/32 (50.0%)*	7/7 (100%)**	12/15 (80.0%)**
ALF	9/18 (50.0%)*	3/7 (42.3%)*	0/7 (0)	4/17 (23.5%)**	0/3 (0)	2/3 (66.7%)*

A. EBV/CMV primary infection with multiple pathogens. B. EBV/CMV primary infection with a single or no other pathogen. C. EBV/CMV past infection with multiple pathogens. D. EBV/CMV past infection with a single or no other pathogen. E. EBV/CMV-uninfected children with multiple pathogens. F. EBV/CMV-uninfected children with a single or no other pathogen. Between * and **, the *p *value < 0.05. H. stay: hospital stay; D. of fever: duration of fever; ALC: atypical lymphocytes; ESR: erythrocyte sedimentation rate; CRP: C-reactive protein; ALF: abnormal liver function (alanine aminotransferase or aspartate aminotransferase higher than 46 U/L); WBC: white blood cell.

**Figure 3:**
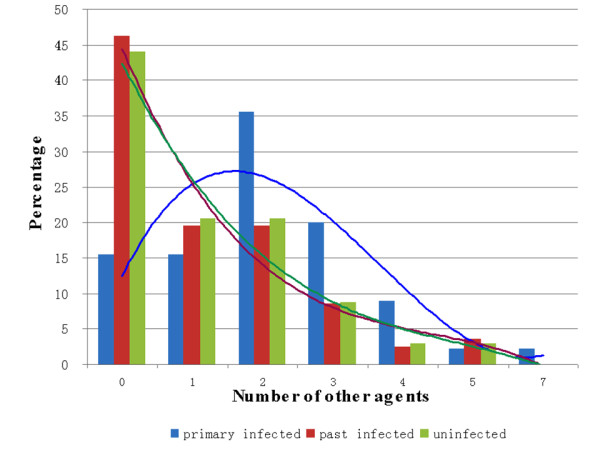
**Correlations between the percentage of patients and the number of pathogens in children with multiple infections**.

Fig. 3 shows that in the primary-infection group, coinfection with two or three pathogens was most frequent, with the percentage first increasing then decreasing when the number of pathogens was more than two. In this group, up to seven pathogens were detected in individual patients. The incidence of coinfection decreased with the number of pathogens in past-infected and uninfected children. In the primary-infection group, the most frequent combination was coinfection of EBV/CMV with two other agents, while one episode involved coinfection with five agents and one episode involved coinfection with seven agents.

The distribution of the 12 pathogens in the multiply infected patients is presented in Table 6. Overall, the most frequent pathogens in the EBV/CMV primary infection group were Flu A and Flu B, followed by CP. In the past-infected group, *K. pneumoniae *was most frequent, and MP was most frequent in EBV/CMV-uninfected children. The incidence of RSV, ADV, MP, Flu A, PIV 1 and PIV 2 did not differ between EBV/CMV-uninfected children and those with primary or past infection. The incidence of CP in the primary-infection group was significantly higher than in the other groups (*p *< 0.001). There was a significantly higher proportion of Flu B (*p *= 0.003) in uninfected children than in the other groups. In the primary-infection and uninfected groups, the proportion infected with PIV 3 was the same and was significantly higher than in children with past EBV/CMV infection (*p *= 0.014). *H. influenzae *was more frequent in the past-infected group compared with the primary-infection group, but did not differ compared with the uninfected group. The incidence of *K. pneumoniae *in past-infected children was significantly higher than that in uninfected patients or those with primary infection (*p *< 0.001).

**Table 6:** The distribution of the other 12 pathogens in multiply infected children.

	primary infected	past infected	uninfected
RSV	9/31 (29.0)	6/28 (21.4)	2/12 (16.7)
ADV	11/31 (35.5)	11/28 (39.3)	3/12 (25.0)
CP	16/31 (51.6)**	7/28 (25.0)*	3/12 (25.0)*
MP	15/31 (48.4)	12/28 (42.9)	4/12 (33.3)
Flu A	12/20 (60.0)	13/18 (72.2)	6/10 (60.0)
Flu B	12/20 (60.0)*	11/18 (61.1)*	8/10 (80.0)**
PIV 1	2/20 (10.0)	2/18 (7.14)	0/10 (0)
PIV 2	1/20 (5.00)	1/18 (3.57)	0/10 (0)
PIV 3	4/20 (20.0)*	2/18 (7.14) **	2/10 (20.0)*
*H. influenzae*	1/20 (5.00)*	3/18 (16.7)**	1/10 (10.0)
*K. pneumoniae*	4/20 (20.0)*	8/18 (44.4)**	2/10 (20.0)*
*L. pneumophila*	0/20 (0)	0/18 (0)	1/10 (10.0)

Between * and **, the *p *value < 0.05. RSV: respiratory syncytial virus; Adv: adenovirus; Flu: influenza virus; PIV: parainfluenza virus; CP: *Chlamydia pneumoniae*; MP: *Mycoplasma pneumoniae*; *H. influenzae*: *Haemophilus influenzae*; *K. pneumoniae*: *Klebsiella pneumoniae*; *L. pneumophila*: *Legionella pneumophila*.

## Discussion

EBV and CMV, members of the herpesvirus family, establish lifelong latent infection. More than 90% of adults have acquired these two viruses [2]. Infants from families of lower socioeconomic levels tend to become infected somewhat earlier than those from better-situated families. In developed countries, primary EBV infection can often be delayed to occur in adolescents and young adults, while in developing countries the prevalence of IgG antibodies to VCA of EBV can be up to 80% by the age of five years without detectable symptoms being reported[15]. In this study there were only 63 children (38.4%) under six years old with EBV primary or past infection. The reason why this percentage is much lower than that in previous studies may be that the objects selected for this study presented with some symptoms of IM. Some authors have noted that maternal antibodies to EBV, most of which disappear by four months of age, may serve to prevent the infection during early infancy [2]. EBV primary infection can occur in infants 2-3 months after the disappearance of maternal antibody [16], meaning that EBV primary infection may occur in infants at six months of age. A study in 2001 in Hong Kong found that the earliest appearance of EBV primary infection occurred in some babies at eight months of age[2], while in our study the youngest infant with EBV past infection (positive for both VCA-IgG and EBNA-IgG) was only two months of age. This should rule out the possibility of protection from maternal antibodies to EBV-VCA and EBNA.

The defensive responses to infection with EBV/CMV can be limited or very broad, which leads to diverse clinical manifestations of infection. The majority of patients with primary infections are usually asymptomatic, except for the acute infectious mononucleosis that is most common in China in children in the 3-6 years age group [15]. Our results showed that the only significant differences in patients with EBV primary infection compared with those having past infection or no infection were a higher incidence of lymphadenopathy and longer hospital stays. The patients in the CMV primary-infection group had longer hospital stays and higher frequency of palatal petechiae, hepatomegaly, splenomegaly, atypical lymphocytes > 10% and abnormal liver function, but fewer rashes than the other two groups. This suggested that the differences in clinical features among the CMV-infected groups occurred much earlier than those among the EBV-infected groups. In addition, in this study seven children showed both EBV and CMV primary infection. They all presented with the typical manifestations of IM and with a high occurrence of hepatomegaly (57.1%), splenomegaly (57.1%) and liver function abnormalities (80.0%). The rate of coinfection with other pathogens was as high as 100% (5/5), and the prevalence of multi-pathogen infection was up to 80% (4/5), which was higher than that of the children with a single EBV or CMV infection. Some authors have reported cases of children with both EBV and CMV infection and noted that the course of disease in these children was longer, but the last word is not yet in on whether coinfection with both EBV and CMV can cause other more serious clinical manifestations[8,9].

The disease spectrum of EBV/CMV primary infection is very diverse, with the most common manifestation being IM. In most studies published outside China, about 50% of children with EBV infection develop IM [17], and the proportion of IM seen in our study was similar (52.5%), which is much higher than other studies in China. In most Chinese studies, the proportion of IM in the disease spectrum is only about 20%, and the most common effect is respiratory tract infection (about 40% compared with 30% in our study)[15]. The disease spectrum of EBV infection is more diverse than that of CMV infection. In addition to IM and respiratory tract infection, Kawasaki disease, anaphylactic purpura, idiopathic thrombocytopenic purpura, measles, asthma, juvenile rheumatoid arthritis and other complications have been reported. Other diseases have also been reported including viral encephalitis, facial paralysis, myocarditis, lymphoma, hemophagocytic syndrome and systemic lupus erythematosus[15]. The complexity of the manifestations and the variety of the disease spectrum of EBV/CMV primary infection suggest that our pediatricians should make the diagnosis based on a comprehensive analysis.

The notable finding in our study was the presence of coinfection of multiple other agents with EBV/CMV in more than 60% of the children. In the groups with detectable CMV antibodies without EBV, this proportion was as high as 81.3%. The most frequent combination was coinfection with two agents. Research on multiple infections accompanying EBV/CMV infection is relatively rare. The prevalence of mixed infection in previous studies is lower than 10% in young children with IM, with the most frequent combination being coinfection with two other pathogens [12]. In contrast, we found a much higher incidence of coinfection with more than two agents.

The differences in the incidence of coinfection may be due to the different types of etiological agents involved or to the different diagnostic methods applied [18,19]. All of the 12 respiratory pathogens detected in our study are active in cold and dry environments. It is possible that these agents would be associated with EBV/CMV because they circulate most frequently at the same time of year [20]. The use of the IIF method to detect antibodies to respiratory pathogens may be another cause of the higher rate of coinfection in our study. IIF is only a qualitative method to detect antibodies, and the existence of IgM antibodies cannot guarantee that the child was infected with multiple pathogens at the same time. In most studies, IgM antibodies can be detected in more than 70% of children with an acute respiratory tract infection within one week of onset of infection, after which the IgM level gradually declines and becomes undetectable three months after the onset of infection. Thus, the IIF method to detect antibodies may merely indicate that a child has been infected with a respiratory pathogen between one week and three months before the sample was obtained [14].

In the patients with multipathogen infections, EBV/CMV may be a primary, co-primary, or secondary pathogen. It may be reactivated in the course of infection with another agent or, possibly, it may precipitate infection with some other organism by suppressing immune function. We prefer the latter hypothesis. Transient immunosuppression secondary to EBV/CMV infection has been well described. During the early phase of acute EBV-related IM, dramatic antigen-driven clonal expansions of CD8 T lymphocytes with an abnormally low CD4+/CD8+ ratio were detected [21-23]. Furthermore, B-cell function was impaired and the production of antibody against other pathogens was inhibited [24,25], but these abnormalities disappeared during the convalescent phase. This demonstrates that infection with EBV can affect both cell-mediated and humoral immunity, and causes a broad-based transient immunosuppression. This immunosuppression may be severe enough to cause secondary infections in some EBV-infected individuals, as illustrated by the report of severe measles and severe RSV pneumonia in patients infected with EBV [10,13,26]. However, whether it is the EBV/CMV infection that causes a mixed infection, or whether the EBV/CMV infection coexists with these diseases is worthy of further exploration.

In this study, the symptoms and physical signs seemed to be most severe in the patients with EBV/CMV primary infection and multiple pathogens. Although there are no similar reports, patients coinfected with EBV/CMV and a single other pathogen such as CP or RSV were reported to suffer more severe symptoms [10,11]. In the multiply infected patients, the distribution of the 12 additional pathogens is not random (Table 6). Coinfection with certain pathogens occurs more frequently than expected in the patients with EBV/CMV primary infection: CP and PIV 3 were more frequently seen and in contrast, all three bacteria were rarer. There have been no previous reports of similar findings.

In conclusion, we found frequent multipathogen infections in children admitted with EBV/CMV infection, and the distribution of these pathogens was not random. Despite this, because most of the children with coinfection of EBV/CMV and multiple pathogens are severely affected, the diagnosis is very important to make. Further studies are needed to clarify the pathogenesis and interactions involved in coinfection by different pathogens.

### Study Design

#### Case selection

One hundred and ninety patients, including 120 boys and 70 girls with ages ranging from 1-164 months (mean 43.5 ± 35.4 months), were enrolled for the retrospective study. All were admitted to Zhongnan Hospital of Wuhan University, China, between August 2008 and September 2009 with suspected IM because they presented with either (1) at least three of the EBV-related symptoms of fever, rash, lymphadenopathy, pharyngitis, palatal petechiae, hepatomegaly, or splenomegaly, or (2) fever of duration longer than seven days. In addition, all EBV-associated malignant diseases such as malignant lymphoma and chronic active EBV infection were excluded.

#### Case definition

##### EBV-infected patients

Primary infection: presence of IgM to viral capsid antigen (VCA) is conventionally used for diagnosing acute EBV infection. However, VCA-IgM is usually transient and quickly disappears, and the test may not be sufficiently sensitive [27-30]. Therefore, in our study, we used an alternative approach to define primary EBV infection as detection of either positive IgG to the early antigen (EA) or low-affinity anti-VCA-IgG or both.

Past infection: positive for IgG to VCA and IgG to Epstein-Barr nuclear antigen (EBNA), or detection of high-affinity anti-VCA-IgG without VCA-IgM and EA-IgG.

Uninfected: no antibodies to EBV detected.

##### CMV-infected patients

Primary infection: positive for CMV-IgM.

Past infection: detection of CMV-IgG without CMV-IgM.

Uninfected: no antibodies to CMV detected [31].

#### Procedures

In this study, a peripheral blood sample was obtained from all children within the first 24 h of admission to the pediatric department. Specific antibodies to EBV and CMV (IgM and IgG to VCA, IgG to EA and EBNA of EBV, IgM and IgG to CMV) were detected by indirect immunofluorescence (IIF). Ninety-three children had an additional test for the affinity of IgG against VCA of EBV (EUROIMMUN, Lübeck, Germany). Moreover, specific antibodies (IgM, IgG) to another 12 respiratory pathogens (respiratory syncytial virus (RSV), adenovirus (Adv), influenza virus (Flu) types A and B, parainfluenza virus (PIV) types 1, 2, and 3, *Chlamydia pneumoniae *(CP) and *Mycoplasma pneumoniae *(MP), *Haemophilus influenzae*, *Klebsiella pneumoniae *and *Legionella pneumophila*) were detected using a commercial indirect immunofluorescence (IIF) kit (EUROIMMUN, Lübeck, Germany) following the manufacturer's instructions.

For each patient, the medical history, age of onset, forewarning signs, symptoms, complications and laboratory data at diagnosis were collected and analyzed.

### Statistical analysis

General data are presented as the percentage or mean ± standard deviation (SD). All statistical analyses were performed using SPSS software (version 13; Chicago, IL, USA). The chi-square test was used to compare between-group differences in percentages. The differences among the mean values of white blood cell counts, hemoglobin and platelets were analyzed using a one-way ANOVA. *p *< 0.05 was considered significant.

## Abbreviations

EBV: Epstein-Barr virus; CMV: Cytomegalovirus; RSV: respiratory syncytial virus; Adv: adenovirus; Flu: influenza virus; PIV: parainfluenza virus; CP: *chlamydia pneumoniae*; MP: *mycoplasma pneumoniae*; IM: infectious mononucleosis; VCA: viral capsid antigen; EA: early antigen; EBNA: Epstein-Barr nuclear antigen; IIF: indirect immunofluorescence

## Competing interests

The authors declare that they have no competing interests.

## Authors' contributions

XW wrote the manuscript and collected the data; KY, CW, YH discussed and reviewed the manuscript. DZ designed the manuscript and analyzed the data; all authors read and approved the final manuscript.
